# Personality development in non-domesticated house mice: evidence for a nutrition-dependent sensitive period early in life

**DOI:** 10.1098/rspb.2024.2689

**Published:** 2025-07-02

**Authors:** Nicole Walasek, Milan Jovicic, Anja Guenther

**Affiliations:** ^1^ University of Amsterdam, Institute for Biodiversity and Ecosystem Dynamics, The Netherlands; ^2^ Max Planck Institute for Evolutionary Biology, Germany; ^3^ University of Hildesheim, Department of Biology, Germany

**Keywords:** *Mus musculus domesticus*, phenotypic plasticity, stress coping, nutrition, animal personality

## Abstract

Changing nutritional conditions pose challenges to developing organisms. Animals typically cope with such changes by adjusting their foraging, exploration or risk-taking behaviours. Notably, there exists considerable between-individual variation (‘animal personality’) in coping strategies. One dimension underlying such personality differences is whether animals cope actively or passively with stress. At present, we know little about how stress coping develops. Previous research found that house mice can adjust stress coping to food quality changes within three generations. However, understanding when during development such personality adjustments occur is crucial for understanding population dynamics in the wild. We tested experimentally how changes in food quality at different ontogenetic stages (fetus, newborn, weanling and late adolescent) affect personality development in non-domesticated cage-housed house mice (*Mus musculus domesticus*). Personality traits were assessed in the open field and the elevated plus maze at different ages (weaning, early adolescence, late adolescence and adulthood). We highlight two key findings. First, the fetal life stage is a sensitive period for stress coping in response to experiencing decreases in nutritional quality. Second, experiencing an increase in nutritional quality may slow the age-related switch towards a passive stress-coping strategy. Our study contributes towards understanding the complex relationships between development, nutrition and personality.

## 1. Introduction

Changes in food availability or nutritional quality can have drastic consequences for organisms’ development. Nutrition across ontogeny is an important determinant of both physiological and behavioural development: The quality, abundance and timing of available food determine when and how much organisms can grow, which may shape their mating and reproductive strategies [1–6]. For example, in bulb mites (*Rhizoglyphus robini*), nutritional conditions during the final developmental stages strongly determine whether males mature as aggressive and strong fighters or benign and weak scramblers [7,8]. In consuming food, organisms not only experience physiological changes but also learn about their environment [9]. These changes in the physiological and information state can in turn elicit changes in behaviour, which may then amplify initial differences in state. For example, stronger or higher-ranking individuals may be more likely to engage in aggressive encounters, which can further increase their strength and rank. Such feedback loops between an animal’s state (physiological and informational) and behaviour may underlie the development of consistent individual differences [10,11]. Other, not mutually exclusive, explanations include life-history trade-offs and fluctuating selection as evolutionary drivers, or developmental noise as an alternative proximate mechanism [12–14]. Resulting behavioural differences that are consistent across time and contexts (statistically indicated by being repeatable) are typically referred to as ‘animal personality’ [15–17].

Over the past decade, interest has grown in understanding how changes in nutritional conditions shape animal personality [18–22]. A recent meta-analysis suggests that poor nutritional conditions tend to be associated with greater risk-taking behaviour—implying lower levels of risk aversion—across various species [21]. For example, mustard leaf beetles (*Phaedon cochleariae*) reared on low-quality food demonstrate greater risk-taking as adults compared to beetles who received high-quality (HQ) food [23]. Such increases in risk-taking may increase foraging success at the expense of increased predation risk. Another example in Argentine ants shows that the willingness to take foraging risks depends on individuals’ nutritional needs [24]. These examples illustrate how trade-offs between nutritional needs, predation risk and potential foraging success come together to shape behaviour and, potentially, to maintain among-individual differences.

One important dimension underlying these differences in risk-taking is how animals cope with stress. Studies commonly distinguish between active and passive stress coping strategies [25,26]. For example, individuals who are bolder and display higher risk-taking tendencies are considered to actively cope with stress [27,28]. Various factors are known to shape coping behaviours, such as parental effects, social context or nutrition, and studies recognize that these factors can have an especially strong influence at early life stages, including the fetal stage [29]. However, existing studies rarely rigorously compare different ontogenetic windows both for the exposure to a specific experience and for measuring its effect [30]. Thus, although developmental processes are recognized as an important factor for understanding animal personality [30–32], we know little about how consistent differences in stress coping develop.

Experimental work in mice has provided insights into the development of stress coping. A study in African striped mice (*Rhabdomys dilectus chakae*) found that protein deficiency experienced either pre- or postnatally increased adult anxiety and passive stress coping [33]. Two recent studies in wild-caught non-domesticated house mice (*Mus musculus domesticus*) explored how changes in nutritional quality shape stress-coping behaviours and life-history traits of mice living in semi-natural enclosures [34,35]. Prabh *et al.* [35] showed that mice can adjust life-history traits and behaviours within just three generations of experiencing changes in nutrition. Mice that underwent a switch towards calorie-dense, HQ food showed higher fecundity and shorter generation time, as well as more passive stress-coping compared to mice fed with standard-quality (SQ) food. These differences became more pronounced across generations. In a subsequent study in the same population of mice, Lopez-Hervas *et al.* [34] shed light on the within- and trans-generational processes underlying these long-term adaptations to changes in nutritional conditions. The authors found that adult mice can flexibly adjust life-history traits (reproduction, weight and growth rates but not survival) to changes in food quality but not stress-coping behaviours. Irrespective of food quality, offspring of parents who experienced a food switch developed more active stress-coping strategies and slower growth rates compared to mice experiencing stable nutritional conditions.

Taken together, these studies suggest that adult mice can adjust life history, but not stress-coping traits in response to changing nutritional conditions. This implies that the window of plasticity for modulating stress-coping has closed before adulthood. Developmental windows during which experiences exert the largest effect on development are called ‘sensitive periods’ [36–38]. It remains an open question when during ontogeny changes in nutritional conditions have the largest effect on stress coping in rodents.

The present study provides insights into sensitive periods for stress-coping in response to changing nutritional conditions in non-domesticated house mice (*Mus musculus domesticus*). We experimentally manipulated the timing of changes in food quality in 455 cage-housed mice and measured its effect on stress-coping behaviours across ontogeny. Stress coping was measured at four time points during ontogeny in an open field and an elevated plus maze. Measurements, in these forced exploration tasks, predict stress-coping behaviours in mice under ecologically relevant conditions [28,39]. We collected data in ‘control mice’ (never experienced a food switch) and in ‘treatment mice’ (experienced a food switch at different times during ontogeny). Ultimately, we sought to answer the following research questions: First, how does stress coping change as a function of food quality across ontogeny? Second, when during ontogeny is stress coping in mice most sensitive towards a food switch? We derive hypotheses for these research questions based on previous studies [34,35].

Regarding the first research question, we expect mice continuously fed with HQ food to display a higher tendency for passive stress coping compared to mice continuously fed SQ food. This prediction is derived from previous work [34,35] and leans on asset-protection theory: Mice fed with HQ food have reproductive value (their ‘asset’) to lose and should avoid risks [13,14,34,40]. We expect cumulative effects of diet, such that effects should be more pronounced in older mice who experienced the diet for longer. We do not have prior expectations about whether food type moderates the relationship between age and stress coping.

Regarding the second research question, we do not expect a food switch in older mice, approaching adulthood, to modulate behaviour. Previous work has shown that adult mice do not exhibit behavioural plasticity in stress coping [34]. Such diminished or absent plasticity in adulthood is common for many traits; it may result from trade-offs between plasticity and phenotypic specializations, or physiological maintenance costs [38,41]. However, we expect that a food switch earlier in ontogeny (*in utero*, at birth, or at weaning) modulates behaviour [29,33]. We do not have prior expectations about which of these ontogenetic time points exerts the strongest effect. As with the first research question, we expect that behavioural consequences of a food switch are more pronounced in individuals who experienced the new diet for longer.

## 2. Methods

### (a) Animals and housing

Our mice are laboratory-reared descendants of wild-caught house mice (*Mus musculus domesticus*) from the Cologne/Bonn region of Germany. Outbreeding and the introduction of fresh caught individuals maintained genetic diversity within our facility. For breeding, we caught young adult animals stemming from replicate semi-natural populations that received either SQ food (Altromin 1324), containing a total of 3227 kcal kg^−1^ metabolizable energy (24% protein, 11% fat, 65% carbohydrates) or HQ food (Altromin 1414), containing approximately 12% more metabolizable energy, i.e. 3680 kcal kg^−1^ (28% protein, 22% fat, 50% carbohydrates) for five generations. Such HQ food, typically used for breeding, has been shown to increase reproduction and growth rate without making mice obese [34,35]. Semi-natural enclosures were built to mimic naturally occurring conditions of wild house mice [34], for details see [33].

After removal from the semi-natural enclosures, we let our mice habituate to being housed in a cage for at least three months in same-sex pairs before establishing breeding pairs. Mice continued to receive the same food *ad libitum* as in the previous generations (i.e. SQ or HQ food). All mice used in this study are direct descendants of these mice and were born and raised in cages. Breeding cages were 425 × 265 × 180 mm to facilitate the building of species-typical nests, and housing cages were 425 × 265 × 150 mm. Cages were equipped with woodchips for bedding, nesting materials, two shelters and various enrichment items (e.g. a running wheel, a seesaw or climbing materials), which were changed every two to three weeks. Light and temperature varied naturally, but underfloor heating prevented temperatures to fall below 10°C. Throughout the experiments, animals were weaned at 4 weeks of age. Thereafter, they were kept in same-sex family pairs. To allow individual recognition, all animals were marked with a subcutaneously implanted RFID chip (Planet ID, ISO, 1.4 × 9 mm).

### (c) Data collection

The data have been collected according to standard protocols used in the open field and elevated plus maze test (details are provided below). Videos were recorded and analysed using Ethovision (Noldus XT 16) software.

All animals had food available *ad libitum*. All experiments were conducted between 06.00 and 10.00 (i.e. at the end of the activity period of mice). Thus, all animals had enough time to feed prior to experiments. In addition, experiments only lasted for 5 min such that hunger would not directly influence the outcome. Only one individual per cage was tested per day, and each animal only conducted one test per day to avoid any carry-over effects. Cages were transported individually to the testing room immediately prior to the test. All mice were handled by tunnel handling to reduce stress of capture prior to the test. The test setups were cleaned with 70% ethanol between trials. Light conditions during the test were constant at 200 lux. All animals were handled only by experienced experimenters.

### (d) Behavioural tests

Stress-coping and stress perception were measured during 5 min long tests in the open field and elevated plus maze. Both tests assume different levels of perceived risk in different arena parts. The open field consists of a 60 × 60 cm brightly illuminated arena, which quantifies the time spent in the central area (number of minutes relative to total test time, as a percentage) and total distance covered (in cm). The former indicates how stressful this environment was perceived, with shorter durations in the centre indicating higher stress levels. The latter indicates how mice cope with this stress-inducing experience. Specifically, larger distances indicate active coping, while shorter distances indicate passive coping.

The elevated plus maze, which consists of a cross-shaped maze with two open, bright arms and two enclosed, dark arms, is less stressful compared to the open field [42,43]. The two bright arms oppose each other and so do the two dark arms. For the elevated plus maze, we recorded the following behaviours: time spent in the bright arms (number of minutes relative to total test time, as a percentage) and the total distance covered (in cm). As with the open field, the proportion of time spent in the open arms of the maze (excluding the centre area between arms) indicates how stressful this environment was perceived. Shorter amounts of time spent in this area indicate that the environment was perceived as more stressful. As in the open field, the distance covered in the maze is indicative of the stress-coping strategy with larger distances indicating active coping.

These tests were originally developed to study rodents’ responses to challenging and potentially threatening situations [44]. They leverage that rodents exhibit thigmotaxis, meaning that they avoid open areas and prefer to stay in close contact with the walls of the apparatus. In mice, measurements from these tests have been well validated in laboratory strains but also linked with ecological parameters for wild-caught individuals [28,39].

### (e) Experimental design

The experimental design comprises four individual experiments, each introducing a food switch at a different time point in ontogeny (electronic supplementary material, figure A1). Across the four experiments mice experienced a food switch during the fetal stage (mother’s third trimester; experiment I), at birth (experiment II), at weaning (experiment III) or in late adolescence (corresponding roughly to early adulthood; experiment IV). Once a food switch occurred, mice remained on this diet for the remainder of the experiment. Mice could be switched from SQ to HQ food and vice versa. For comparative purposes, we maintained two control groups of mice who have been continuously fed SQ or HQ food. In line with previous work [34,35], we contrast SQ and HQ food to examine the effect of food quality changes on behavioural phenotypes beyond reproduction. We were not interested in the effects of nutritional deprivation and thus did not consider low-quality food.

**Figure 1.**
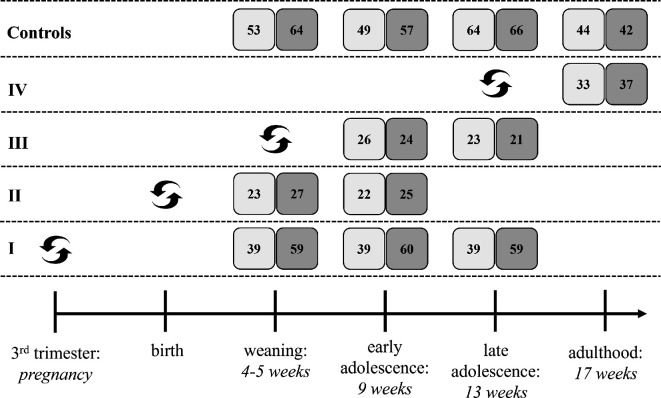
Overview of the food switch and measurement times across experiments. This figure illustrates when mice within each experiment (rows) were tested. Timing of food switch is indicated by the black, curved arrows and measurement times are indicated by grey squares. The top row shows measurement times in control mice. The numbers within each square indicate the number of mice that underwent a specific experiment (indicated by the row) and were measured at a specific time (indicated by the horizontal axis). Light grey squares show sample sizes of mice who started on standard-quality (SQ) food, and dark grey squares correspond to mice who started on high-quality (HQ) food. We report the final sample sizes from the open field experiment. The table shows a relatively large difference in sample sizes between SQ and HQ fed mice who experienced a food switch during the mother’s pregnancy (experiment I). This difference results from reduced fecundity, reduced litter size and higher offspring mortality of mothers fed with SQ food compared to mothers fed with HQ food [35].

Figure 1 illustrates when in ontogeny mice were tested during each experiment. Each mouse was always first tested in the open field and then in the elevated plus maze at least 48 h later. Testing only started at weaning after the mice gained independence from their mothers. At this time, physiological and neurochemical development has completed to allow fully functional predator avoidance. Mice were tested between four and six weeks after the food switch, and each subsequent test occurred at roughly four week intervals (figure 1). A recent experiment shows that this interval is sufficiently long for mice to ‘forget’ the previous test [45]. We did not test behaviour right before or after a food switch as our primary interest lies in the long-term development of behavioural differences following food switches.

Within experiments mice have been assessed repeatedly between 1 and 3 times at different test ages (see figure 1). Mice have not been tested multiple times per test age. In the control conditions, we replaced mice for different experiments such that control mice are not tested more often than treatment mice. This results in a fluctuating sample size of control mice (figure 1).

### (f) Dataset and variables

The final dataset consists of 455 cage-housed mice (215 females) with a total of 1142 measurements from the open field and 1122 from the elevated plus maze (EP). The measures were taken across four different test ages (i.e. weaning, early adolescence, late adolescence and adulthood). Of those 1142 (EP: 1122) measures, 423 (EP: 419) are from control individuals who never experienced a food switch and 719 (EP: 703) measures are from the treatment group who experienced a food switch. These numbers already account for missing data (see below).

Aside from information about the food type, timing of food switch and test age, additional variables indicate the sex of the mouse, mother ID and cage ID.

### (g) Missing data and outliers

Twelve cases had missing cage ID values due to manual data entry errors; we assigned unique IDs to these. We excluded 2 cases with missing open field outcome measures and 22 with missing elevated plus maze outcomes from their respective analyses. Missing values stemmed from mice jumping out of the apparatus (*n* = 3) or separation due to aggression between cage mates, causing both mice to drop out. Stress from the open field test, which always preceded the elevated plus maze, may have triggered this aggression, explaining higher missingness in the latter test. No other variables had missing data.

Before analysis, we excluded 8 elevated plus maze cases where the tracking software failed to locate the mouse for over 20% of the time. Wild mice can jump up to 70 cm high and wide, often causing the camera to lose them, especially in the plus maze with its corners and walls. This did not occur in the open field, so all measures there were retained.

We excluded 3 statistical outliers when analysing mice who were switched from HQ to SQ food as adults (experiment IV): We removed data points that lay outside four times the interquartile range to improve model fit (electronic supplementary material, figures A6–A9). In these 3 trials, three different adult mice who were continuously fed HQ food reached extreme distances of over 9000 cm in the open field—a potential indicator of rare hyperactivity. Our results are qualitatively identical with and without these data points.

### (h) Statistical analyses

All analyses were performed in R (v. 4.4.1). We computed repeatabilities of all four behavioural measures adjusted for age using the *rptR* package (v. 0.9.22) [46]. To account for the nesting and repeated-measures structure in our data, we fitted mixed-effects models from the *lme4* package (v. 1.1.35.5) [47]. We assumed Gaussian distributions of all outcome measures. We used the *performance* [48] package (v. 0.13.0) to visually check assumptions and model fit (i.e. normality of residuals, linearity and homogeneity of variance). The variable indicating time spent in the centre of the open field (centre OF) was square-root transformed to approximate a Gaussian distribution and improve model fit. We used the packages *lmerTest* (v. 3.1.3) [49] and *pbkrtest* (v. 0.5.3) [50] to approximate Kenward–Roger degrees of freedom for statistical inference (i.e. *p*-values and confidence intervals). Additionally, we computed bootstrapped *p*-values and confidence intervals using the package *parameters* (v. 0.24.1) [51] to verify the robustness of our statistical inference. We find that all estimation methods lead to similar results. To control the false-discovery rate (i.e. significant findings that are false positives due to a large number of tests), we use the Benjamin–Hochberg procedure to adjust *p*-values across analyses. For each of our four outcome measures, we ran separate models with the same set of predictors. The *p*-value correction (Benjamin–Hochberg) was applied across these models with different outcome measures. Thus, for each *p*-value of each predictor, we report a ‘corrected’ *p*‐value, adjusted across the four outcome measures.

To estimate the effects of categorical predictors, we conduct analyses of variance (ANOVAs) with Kenward–Roger degrees of freedom based on the mixed models. We estimate type III sums of squares. When our research question requires it, we use the package *emmeans* (v 1.10.3) [52] to perform post hoc comparisons between groups and to estimate marginal means. We apply the Tukey procedure to adjust for multiple testing during post hoc comparisons.

We use two-tailed tests for all analyses with an alpha level of 0.05 and 95% confidence intervals. Below we detail the structure of our models answering different research questions.

For transparency, all our data and code, including a pre-registration are available on the Open Science Framework [53].

#### (i) RQ1: How does behaviour change as a function of food quality across development?

This analysis includes only control mice. We perform an interaction (or moderation) analysis with food type as the moderator for each behavioural outcome [54]. We test whether food type moderates the relationship between test age and the outcome while controlling for sex. We use nested random intercepts to account for the nesting of mice in cages and cages in families (i.e. random-effect structure: family/cage/mouse). Due to their involvement in interactions, we use sum-to-zero coding for food type and test age. Here, and in all other analyses, we use dummy coding for sex with females as the reference group. To test for group differences, we conduct post hoc comparisons between food quality and age groups.

#### (ii) RQ2: When during development are mice sensitive towards a food switch?

To ensure that we have a proper control condition for each treatment, we split the analysis into two sets of models: One set compares ‘control mice’ who have been continuously fed SQ food to ‘treatment mice’ who were switched to HQ food. The other set compares mice who have been continuously fed HQ food to mice switched to SQ food. We run separate models for each test age and for each outcome measure. Each model regresses the outcome measure on switch time while controlling for sex. We use nested random intercepts to account for the nesting of cages in families (family/cage). Switch time is dummy coded with never as the reference group. Thus, we do not need additional post hoc comparisons to compare groups.

#### (iii) Exploratory analysis: Does the timing of food switch shape changes in behaviour across development?

Here, we aim to estimate how behaviour changes in developing mice who experienced a food switch. For that purpose, we use mice from different test ages (combined from different experiments) who experienced a food switch at the same times. We combine mice from experiments I and II to estimate changes in behaviour from weaning to early adolescence (possible switch times: birth and pregnancy). We combine mice from experiments I and III to estimate changes from early to late adolescence (possible switch times: pregnancy and weaning). We split the analysis into two sets with separate models for switches from SQ and HQ food (as in RQ2). We perform an interaction (or moderation) analysis with switch time as the moderator for each outcome measure. We test whether switch time moderates the relationship between test age and the behavioural outcome, while controlling for sex. We used nested random intercepts to account for the nesting of mice in cages and cages in families (family/cage/mouse). Switch time and test age were sum-to-zero coded due to their involvement in interaction terms. Significant interactions are followed by post hoc comparisons at each level of the moderator.

### (i) Pre-registration

We pre-registered all analyses carried out in this study as a secondary data pre-registration on the Open Science Framework [55]. We chose the template for secondary data analysis, because the data were already collected at the time of writing the pre-registration. We report the following three minor deviations from our pre-registration. First, contrary to the information in the pre-registration, we used sum-to-zero coding for RQ2 and the exploratory analysis. Using dummy coding, as suggested in the pre-registration, is suboptimal due to the involvement of interaction terms. Second, we suggested an additional exploratory analysis relying on training a classifier to discriminate behaviour between SQ and HQ fed mice. We discarded this analysis because the associations between food type and behaviour were not strong enough to successfully train such a classifier. Third, as described earlier, we had to remove 3 outliers from one analysis to improve model fit. This removal was only based on information obtained from assumption checks (electronic supplementary material, figures A6–A9).

During the reviewing process, we discovered a systematic mislabelling of identifiers of related mice (siblings) during two weeks of the experiment. We were able to recover the correct identifiers for most mice based on the physical experiment logs. In 19 trials (i.e. data rows not mice), we were not able to identify the mouse with certainty and discarded the data. Our results based on the original and corrected data are qualitatively identical. For full transparency, we include both datasets and results derived from them in our code base [56].

## 3. Results

### (a) RQ1: How does behaviour change as a function of food quality across development?

**Figure 2.**
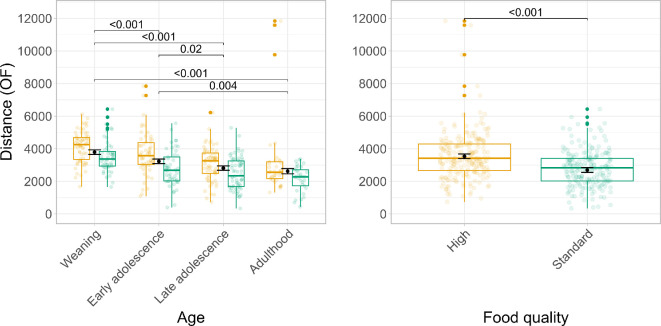
Distance covered in the open field across development. The left panel shows boxplots for distance covered in cm (*y*-axis) across test ages (*x*-axis). Colours indicate the food quality with yellow corresponding to high-quality (HQ) and green to standard-quality (SQ) food. The black points indicate model-based estimated marginal means for each test age and corresponding standard errors. *p*-values indicate significant differences between test ages. The right panel illustrates the main effect of food quality (*x*-axis) on distance covered in the open field (*y*-axis). The black points indicate model-based estimated marginal means for each food type and corresponding standard errors. *p*-values indicate significant differences between food types. *p*-values in both panels have been corrected for multiple post hoc comparisons with the Tukey procedure.

All behaviours were significantly repeatable across age (table 1). Food type did not moderate the relationship between age and any of the behavioural outcomes (electronic supplementary material, tables A1, A2). However, we did find that age significantly predicted all outcomes (distance open field: F _3,297.85_ = 50.51, *p* < 0.001; centre OF: F_3,298.64_ = 9.21, *p* < 0.001; distance elevated plus maze: F_3,285.97_ = 3.09, *p* = 0.028; bright arms elevated plus maze: F_3,301.90_ = 4.82, *p* = 0.004; electronic supplementary material, table A2). Post hoc comparisons (electronic supplementary material, table A3, Figure A3) reveal that time spent in the centre of the open field and in the bright arms of the elevated plus maze increased with age, indicating that older mice perceived the tests as less stressful compared to younger mice. At the same time, older mice covered less distance in both the open field and the elevated plus maze, indicating a switch towards passive stress coping. Across all four behaviours, food type only significantly predicted distance covered in the open field (F_1,78.25_ = 19.25, *p* < 0.0001; figure 2; electronic supplementary material, table A1). As we are particularly interested in the impact of food type on development, we decided to focus on distance covered in the open field as our main behavioural outcome of interest for the remainder of the results. Figure 2 summarizes how distance in the open field changes in relation to age and food type.

**Table 1.** Repeatability adjusted for age. For each outcome, we show repeatability aggregated across food types and separately for each food type. We use the following abbreviations: OF = open field, EP = elevated plus maze, SQ = standard-quality food and HQ = high-quality food. Bold *p*-values highlight significance at an alpha level of 0.05.

repeatability estimates in control mice						
	*R*	*SE*	**2.5% CI**	**97.5% CI**	***p*** **(LRT)**	*p* (permut)
**distance (OF)**	0.370	0.060	0.242	0.472	**<0.001**	**0.001**
SQ	0.691	0.052	0.581	0.774	**<0.001**	**0.001**
HQ	0.201	0.083	0.042	0.364	**0.001**	**0.014**
**centre (OF)**	0.233	0.061	0.117	0.354	**<0.001**	**0.001**
SQ	0.083	0.077	0.000	0.257	0.161	0.160
HQ	0.202	0.081	0.049	0.371	**0.004**	**0.011**
**distance (EP)**	0.411	0.057	0.300	0.520	**<0.001**	**0.001**
SQ	0.438	0.082	0.261	0.587	**<0.001**	**0.001**
HQ	0.383	0.078	0.219	0.536	**<0.001**	**0.001**
**brightarm (EP)**	0.116	0.058	0.000	0.228	**0.029**	**0.024**
SQ	0.016	0.056	0.000	0.190	0.431	0.429
HQ	0.229	0.083	0.065	0.389	**0.003**	**0.003**

### (b) RQ2: When during development are mice sensitive towards a change in food?

**Figure 3.**
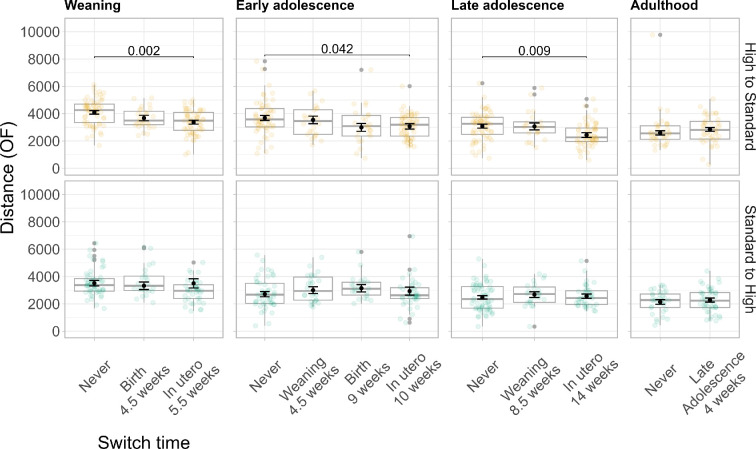
Distance covered in the open field across food switch times. The top row shows results comparing mice who have continuously received high-quality (HQ) food to mice who have been switched from HQ to standard-quality (SQ) food. The bottom row shows results comparing mice who have continuously received SQ food to mice who have been switched from SQ to HQ food. Each column corresponds to one test age. Within a panel the *x*-axis denotes different switch times, with ‘Never’ corresponding to the control group. Beneath each food switch time, the number of weeks indicates how long mice have been fed the new food type. The *y*-axis denotes distance covered in cm. Within a panel boxplots indicate the distribution of the raw data (also shown as lightly coloured circles), while point estimates and error bars (in black) indicate model-based estimated marginal means and standard errors. *p*-values indicate significant differences between mice whose food was switched at different times compared to the control group. *p*-values have been corrected for multiple post hoc comparisons with the Tukey procedure.

Our analysis revealed no significant effects on distance in the open field when switching mice from SQ to HQ food at any of the tested switch times during ontogeny (table 2 and figure 3, bottom row).

**Table 2.** Model summary for modelling distance in the open field at different test ages. All variables were fitted with mixed-effects models if possible. We attempted to fit each model with nested random intercepts with cages nested in families. If the model did not converge, we simplified the random-effects structure (electronic supplementary material, table A18). Degrees of freedom were computed with the Kenward–Roger approximation. Additionally, we computed bootstrapped parameter estimates, confidence intervals and *p*-values. Per model, we applied a correction for multiple testing (FDR correction) to these bootstrapped *p*-values across the four outcome variables that used the same set of predictors. All categorical predictors were dummy coded with the following reference groups: females (for sex) and never (for switch time). Explanation of variables from left to right: starting food: food quality before food switch, *b*: estimated slope, *SE*: standard error, *df*: degrees of freedom, *t*: test statistic, *p*: *p*‐value, *b*: bootstrapped slope, 95% CI: bootstrapped confidence interval around the slope, *p*: p-value associated with bootstrapped estimates, *p* adj.: bootstrapped *p*-value adjusted across models with different outcomes. Bold *p*-values highlight significance at an alpha level of 0.05.

regression output for distance (OF) across test ages										
		Kenward–Roger estimates					bootstrapped estimates			
	starting food	*b*	*SE*	df	*t*	*p*	*b*	**95% CI**	*p*	*p* adj.
**weaning**										
sex (males)	SQ	−65.57	160.70	42.73	−0.41	0.685	−72.16	(−382.087, 245.331)	0.665	0.818
switch time (*in utero*)	SQ	−14.61	385.10	41.98	−0.04	0.970	−22.07	(−764.657, 710.522)	0.952	0.952
switch time (birth)	SQ	−188.14	301.62	60.27	−0.62	0.535	−191.32	(−748.453, 401.452)	0.528	0.528
sex (males)	HQ	−56.45	149.92	141.83	−0.38	0.707	−59.32	(−353.508, 240.955)	0.690	0.942
switch time (*in utero*)	HQ	−714.07	191.32	20.22	−3.73	**0.001**	−712.96	(**−1095.293, −348.138**)	**<0.001**	**0.002**
switch time (birth)	HQ	−413.23	236.45	36.26	−1.75	0.089	−410.50	(−876.21, 43.785)	0.078	0.155
**early adolescence**										
sex (males)	SQ	−184.82	182.35	61.75	−1.01	0.315	−186.65	(−538.422, 171.985)	0.313	0.444
switch time (*in utero*)	SQ	225.28	349.90	26.89	0.64	0.525	230.35	(−442.868, 904.078)	0.508	0.912
switch time (birth)	SQ	433.75	312.66	63.32	1.39	0.170	442.96	(−177.008, 1043.456)	0.164	0.327
switch time (weanling)	SQ	291.96	279.49	79.66	1.04	0.299	290.94	(−266.892, 824.128)	0.300	0.411
sex (males)	HQ	−167.72	193.12	78.54	−0.87	0.388	−168.04	(−540.626, 218.387)	0.400	0.908
switch time (*in utero*)	HQ	−616.61	270.57	25.04	−2.28	**0.031**	−608.89	(**−1134.509, −93.627**)	**0.021**	**0.042**
switch time (birth)	HQ	−692.19	334.05	42.76	−2.07	**0.044**	−690.56	**(−1343.793, −34.482)**	**0.038**	0.150
switch time (weanling)	HQ	−143.79	307.00	91.30	−0.47	0.641	−138.33	(−735.183, 441.148)	0.636	0.870
**late adolescence**										
sex (males)	SQ	−34.03	179.28	62.22	−0.19	0.850	−36.88	(−387.407, 313.607)	0.838	0.838
switch time (*in utero*)	SQ	83.30	201.27	61.03	0.41	0.680	84.46	(−312.485, 474.726)	0.672	0.689
switch time (weanling)	SQ	199.58	244.84	60.77	0.82	0.418	193.94	(−272.672, 682.029)	0.441	0.676
sex (males)	HQ	−154.89	177.64	72.71	−0.87	0.386	−153.09	(−493.373, 195.395)	0.377	0.502
switch time (*in utero*)	HQ	−652.57	226.64	21.35	−2.88	**0.009**	−647.05	(**−1091.716, −197.422**)	**0.004**	**0.009**
switch time (weanling)	HQ	−19.23	276.04	86.24	−0.07	0.945	−17.20	(−549.788, 511.023)	0.946	0.946
**adulthood**										
sex (males)	SQ	−115.49	229.23	44.11	−0.50	0.617	−114.95	(−572.533, 329.864)	0.611	0.611
switch time (adult)	SQ	134.34	228.59	45.45	0.59	0.560	139.98	(−315.415, 571.758)	0.523	0.698
sex (males)	HQ	504.50	204.90	41.83	2.46	**0.018**	503.71	**(104.703, 908.531)**	**0.016**	0.062
switch time (adult)	HQ	250.74	206.48	38.75	1.21	0.232	251.69	(−153.501, 653.512)	0.211	0.295

Our analysis revealed significant effects of switching mice from HQ to SQ food during the mother’s pregnancy, indicating a potential sensitive period *in utero* (table 2 and figure 3, top row). Mice who were switched *in utero* covered less distance in the open field compared to control individuals who continuously received HQ food. This effect was measurable in weanlings (HQ/HQ: *M* = 4128, *SD =* 951; HQ/SQ: *M* = 3540, *SD =* 992), early adolescents (HQ/HQ: *M* = 3706, *SD =* 1296; HQ/SQ: *M* = 3185, *SD =* 1052) and late adolescents (HQ/HQ: *M* = 3082, *SD =* 1029; HQ/SQ: *M* = 2765, *SD =* 1010). Across test ages, we can observe a consistent reduction in estimated distance covered of around 600−700 cm, corresponding to a reduction of 16–20% (table 2). We would like to note that mean differences across all switch-time groups were not significantly different in early adolescence (electronic supplementary material: Table A0). ANOVA tests whether there are significant mean differences across all groups. However, our contrasts in the regression analysis specifically compare each group mean to the reference group (i.e. the control mice) (table 2). Our contrasts thus confirm that early adolescent mice who experienced a food switch during the mother’s pregnancy showed significantly different behaviour compared to control individuals—even though they may not differ significantly from mice switched at other times.

Taken together, these findings suggest that mice are able to adjust their behaviours to a food switch *in utero* when food quality decreases (from HQ to SQ) but not when it increases. This implies a nutrition-dependent sensitive period *in utero*. In the electronic supplementary material, we report results from the same analyses for the other three behavioural outcome measures (electronic supplementary materia: Tables A6–A17).

### (c) Exploratory analysis: Does the timing of food switch shape changes in behaviour across development?

From weaning to early adolescence, the timing of food switch did not shape changes in distance covered in the open field when mice were switched from HQ to SQ food (electronic supplementary material, tables A19, A20). However, switch time significantly shaped changes in distance covered when mice were switched from SQ to HQ food (F_2,107.39_ = 5.92, *p* = 0.015; electronic supplementary material, tables A19, A20). Post hoc comparisons revealed that 13 week old mice (*M =* 2490, *SD =* 995) who consistently received SQ food significantly covered less distance compared to 5 week old mice (*M =* 3546, *SD =* 972; *t*(109.15) = −6.51, *p* < 0.001; electronic supplementary material, tables A21, A22). In contrast, mice who experienced a food switch *in utero* or at birth did not significantly differ across test ages in distance covered (electronic supplementary material, tables A21, A22). We illustrate this relationship in the electronic supplementary material in Figure A4. Our results indicate that switching mice to a more nutritious diet might slow the age-related transition from active to passive stress coping.

From early to late adolescence, switch time did not shape changes in distance covered in the open field, irrespective of diet (electronic supplementary material, tables A23, A24). In the electronic supplementary material, we report results from the same analyses for the other three behavioural outcome measures (electronic supplementary material, tables A19–A24).

### (d) Treatment effects on body mass

To monitor the well-being of our mice, we regularly recorded the body mass of mice in different treatments. In our dataset, we have recorded the body mass in 87% of cases within a week of measuring behaviour (i.e. in the same life stage). We show associations between body mass and our outcome measures in the electronic supplementary material, figure A2. Additionally, we show that the timing of food switches did not impact body mass development (electronic supplementary material, tables A26, A27, figure A5).

## 4. Discussion

### (a) The ontogeny of stress perception and coping

We found that stress perception and coping change across ontogeny in cage-housed mice, even when there is no experimental manipulation. Although older mice perceived the tests as less stressful, they developed increasingly more passive stress coping, indicating higher levels of risk aversion [28]. At the age of 17 weeks, animals in our study expressed comparable trait values to studies investigating stress-coping behaviour in adult house mice living in cages or semi-natural enclosures [35,57]. Understanding the ontogeny of stress-coping lays the foundation for studying how and when experiences can alter developmental trajectories, and when we can measure their effects.

### (b) Nutrition-dependent plasticity

We observed that mice experiencing decreases in nutritional conditions *in utero* adopt passive stress coping, implying higher levels of risk aversion. These effects are measurable in weanlings and across adolescence. Our results align with a vast literature suggesting that prenatal stress exerts long-lasting effects on behaviour in mammals and rodents [58–60]. Mice who prenatally experienced a sudden loss in food quality may adopt risk-averse strategies to manage the uncertainty of future nutritional changes [61].

Our findings dovetail with prior work documenting nutrition dependency in the capacity of mice to adjust stress-coping and life-history traits across one generation [34]. As in our study, this study observed that effects are stronger (or only present) in mice who were switched away from calorie-dense, HQ food. Another related study in African striped mice also observed larger effects of dietary protein reduction compared to increases [33]. These findings suggest that HQ food might function as energetic fuel for plasticity, handing individuals a ‘silver spoon’ for flexible adjustment. A recent study in house mice demonstrating increases in brain volume as a result of an HQ diet lends further support to this explanation [62]. However, contrasting previous work in the same species [34,35], effects of food quality on stress coping in the present study are reversed: mice who receive HQ food show more active stress coping compared to mice receiving SQ food. Below we will discuss the differences between these studies.

Additionally, our data suggest nutrition-dependent effects on age-related changes in stress coping. In our sample, experiencing increases in nutritional conditions appears to slow the age-related transition towards passive stress coping. Mice who were switched to HQ food reduced active stress coping to a lesser extent compared to controls. However, we note that both treatment and control mice have similar trait values in late adolescence. The difference in trajectories appears to be driven by the different starting points: Weanlings who were switched to a HQ diet (*in utero* or at birth) show descriptively lower levels of stress coping compared to controls (figure 3). This difference was not statistically significant. It is unclear whether this descriptive difference reflects a true difference resulting from the treatment or simply noise. Future studies with larger sample sizes are needed to answer this question.

### (c) A sensitive period *in utero*

Our study identifies the fetal life stage—a pivotal developmental period—as a sensitive period for stress-coping behaviours in house mice. Changes in nutritional quality likely shape behavioural differences through their impact on the developing brain [63]. One potential mechanism by which changes in nutritional quality may modulate behaviour is fetal programming [64–68]. The underlying idea is that the mother provides cues, integrating over a lifetime of her experiences, which the developing fetus uses to ‘predict’ the long-term expected nutritional environment [69,70]. Crucially, fetal programming assumes that information provided through the mother better predicts expected nutritional conditions than information sampled postnatally (so-called external predictive adaptive response [71]). This assumption holds more likely when environmental conditions are stable (or autocorrelated) across generations [72]. It can be argued that house mice living in human-made environments, dampening variation in temperature and food availability, experience such stable conditions. However, future studies integrating maternal measures and fitness measures of the offspring are needed to further illuminate the mechanisms at play.

Two limitations of our experimental design warrant caution in drawing certain conclusions about a sensitive period *in utero*. First, we do not have a full-factorial design. For example, we did not measure late adolescent mice who experienced a food switch at birth and we also do not have measures of adult mice who experienced food switches *in utero* or at birth. Second, the number of mice who experienced a food switch at birth is smaller compared to those experiencing a food switch *in utero* (see figure 1). The reason for this is that mothers of mice who were switched from HQ to SQ food at birth often withdrew maternal investment and ate their offspring (see also [33]). Thus, we may not have enough power to detect significant effects of food switches at birth. At least in early adolescence, the descriptive differences between control and treatment mice are similar for the *in utero* and at birth condition. Thus, while our data clearly point towards an early sensitive period for the effects of changes in food quality, we cannot (yet) clearly delineate its timing and duration. Future studies are needed to clearly disentangle whether effects are stronger for food switches *in utero* or at birth.

### (d) Difference between mice living in semi-natural conditions and cage-housed mice

Our study relates to two recent studies exploring how nutritional conditions shape stress-coping and life-history traits in non-domesticated house mice living in semi-natural enclosures [34,35]. In line with asset-protection theory, both studies report that mice receiving HQ food develop passive, risk-averse coping strategies [13,14,34,40]. The idea is that animals adjust their risk-taking behaviour based on their current assets, such as their energy reserves or reproductive value. Consequently, mice fed with HQ food have more to lose and should avoid risky exposure to threats. These findings resonate with a recent study in African clawed frog tadpoles (*Xenopus laevis*) [18]: Food-restricted tadpoles took greater foraging risks in novel environments compared to food-unrestricted tadpoles. However, we observe the opposite pattern in cage-housed mice: Mice receiving HQ food develop more active coping strategies compared to SQ-fed mice. Similarly, caged-housed African striped mice show more active stress-coping on a high-protein diet compared to a low-protein diet [33]. From a theoretical point of view, we could argue that HQ food increases the chances of mice successfully taking risks [73]. A study in hihi (*Notiomystis cincta*) lends support to this idea [74]. It documented weak, positive associations between early-life carotenoid supplementation and boldness, which tends to be positively associated with risk-taking [75].

How can we reconcile these contrasting patterns within the same species and even the same population of mice? One crucial difference between cage- and enclosure-housed mice is the social environment. Cage-housed mice live in same-sex pairs, whereas enclosure-housed mice live in large groups. Thus, cage-housed mice, unable to reproduce, may have more energy to invest into exploration.

However, the social environment is not only important for providing reproductive opportunities. Previous work has highlighted the importance of the social environment for the development of behaviour and personality [76–78]. For example, a series of studies in guinea pigs suggest that males who have been housed in groups during adolescence develop to be less aggressive adults compared to males housed in isolation [79]. Living in social groups might also help individuals to better recover from stressful experiences [80,81]. However, experiencing higher population density may also imply larger competition for resources, making individuals more protective of their assets. Future work could explicitly study the role of the social environment by varying group size in captive and (semi-)free-living mice. While these observed differences across studies suggest that the social environment may moderate stress coping, they also raise concerns about the validity of studies in captive animals—as has been discussed in the literature for a while [82–84]. More work is needed to understand the effects of housing conditions on experimental findings.

Throughout this discussion section, we have suggested several ways in which future work could clarify some of the questions raised by our study. These studies would shed light on the mechanisms underlying stress-coping behaviours in rodents, providing important insights into how and when organisms are able to adapt to changing environmental conditions.

## Data Availability

We uploaded the data and code to the OSF project page [53]. Supplementary material is available online [85].
